# Case Report: Primary segmental volvulus in an infant

**DOI:** 10.3389/fped.2025.1707716

**Published:** 2025-11-13

**Authors:** Wenya Wang, Xiaoping Jiang, Weilu Wu, Li Zhang

**Affiliations:** 1 Department of Pediatrics, West China Second University Hospital, Sichuan University, Chengdu, China; 2 Key Laboratory of Birth Defects and Related Diseases of Women and Children, Sichuan University, Ministry of Education, Chengdu, China; 3 National Health Commission Key Laboratory of Chronobiology, Sichuan University, Chengdu, China; 4 Department of Pediatric Surgery, West China Hospital, Sichuan University, Chengdu, China; 5 Department of Pathology, West China Second University Hospital, Sichuan University, Chengdu, China

**Keywords:** infant, case report, intestinal diseases, volvulus, thrombin-antithrombin complex

## Abstract

Primary segmental volvulus (PSV) is a rare cause of acute abdomen in infants. It is characterized by a form of strangulated intestinal obstruction requiring prompt diagnosis and surgical intervention. This study aimed to report a case of PSV in an infant, which was managed successfully through early recognition, close clinical monitoring, and timely surgical treatment. Although both blood and ascitic fluid cultures were negative postoperatively, metagenomic next-generation sequencing (mNGS) identified the same pathogen in both specimens, enabling targeted antibiotic therapy. This case highlights the importance of including PSV in the differential diagnosis of infants presenting with unexplained abdominal distension and bilious vomiting, particularly when accompanied by anemia. Additionally, the elevated level of the coagulation system biomarker thrombin–antithrombin complex (TAT) may serve as a useful marker for monitoring coagulation status in the perioperative period. The integration of TAT assessment and mNGS-based pathogen identification provides a novel framework for individualized perioperative management in PSV.

## Introduction

Primary segmental volvulus (PSV) is a rare, rapidly progressive, and life-threatening disease that requires early recognition, with surgical intervention being the only effective treatment. Because the clinical manifestations of PSV are nonspecific, the condition is often not definitively diagnosed until laparotomy is performed (1). We found that thrombin-antithrombin complex (TAT) levels may serve as a potential marker for evaluating disease severity and determining surgical indications. In addition, the application of metagenomic next-generation sequencing (mNGS) enables precise pathogen identification and facilitates targeted antibiotic therapy.

## Case report/case presentation

**Figure 1:**
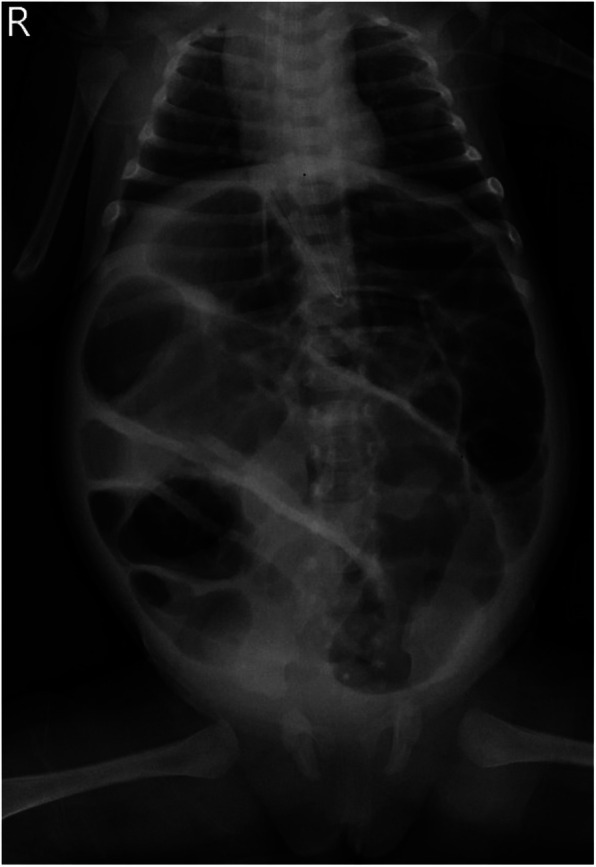
Neonatal abdominothoracic x-ray.

**Figure 2:**
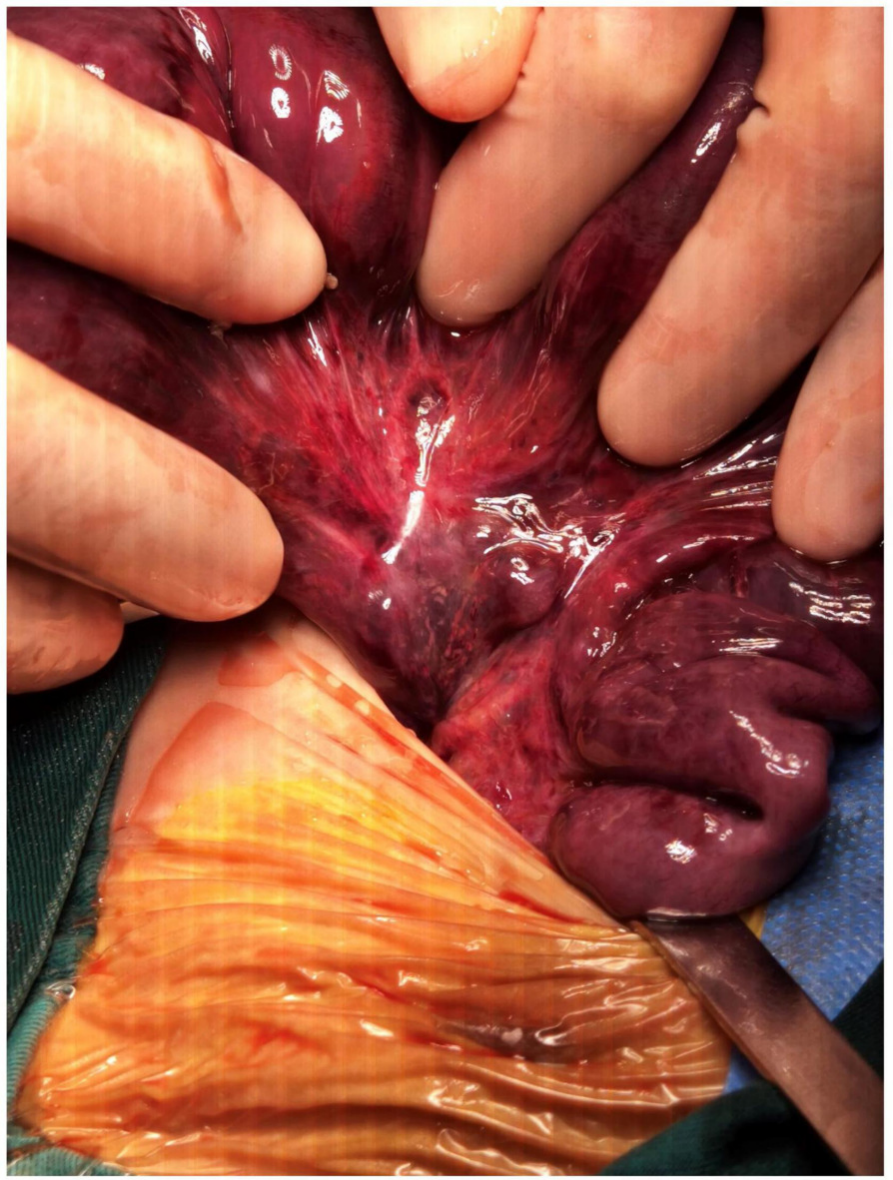
Intraoperative findings.

**Figure 3:**
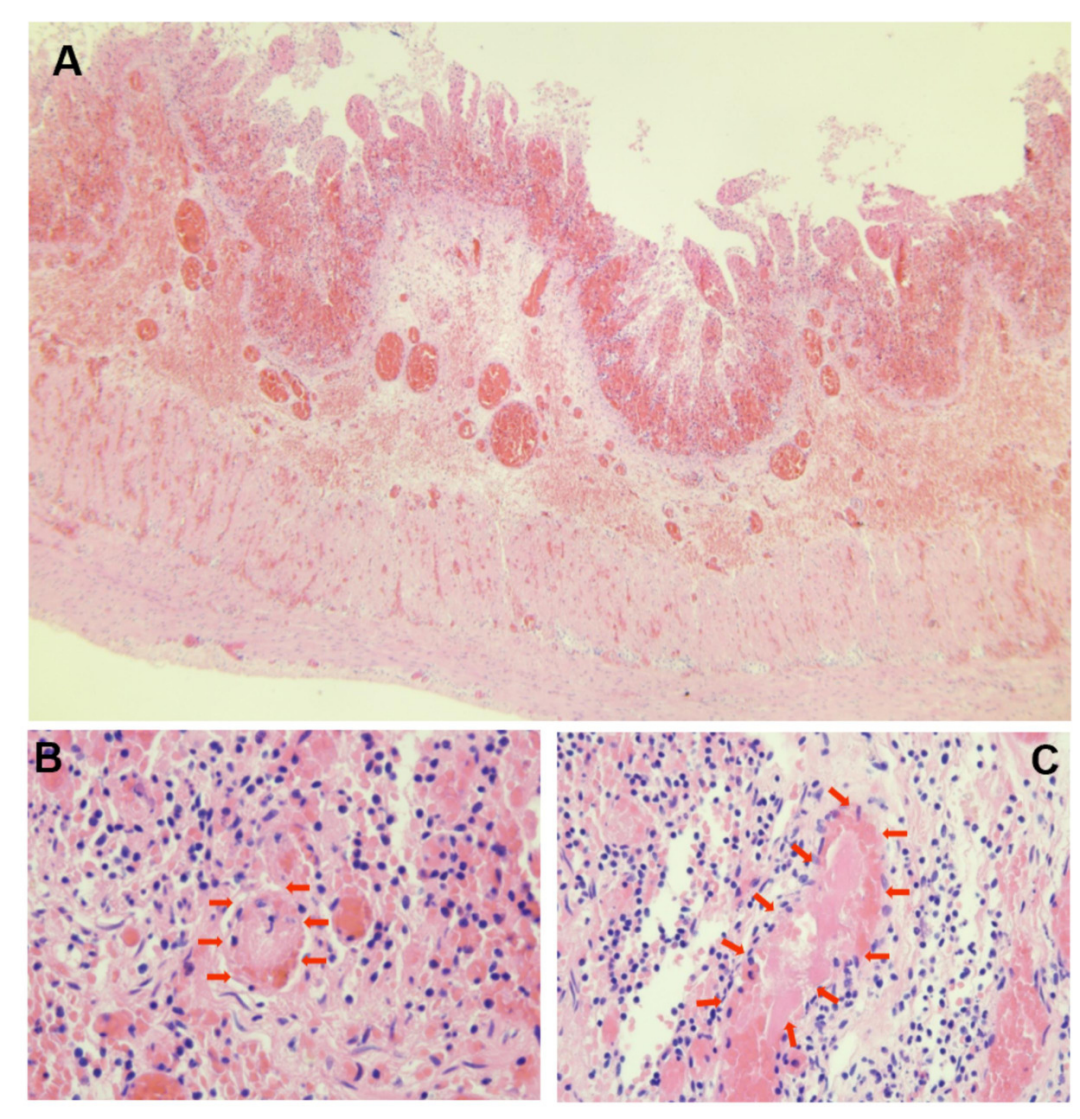
Hematoxylin and eosin–stained sections of necrotic intestine. **(A)** × 40: preserved wall structure with congestion, hemorrhage, and mucosal necrosis. **(B,C)** × 400: red arrows indicating intravascular throm.

A 46-day-old female infant was admitted with a 1-day history of abdominal distension and bilious vomiting. She was born via spontaneous vaginal delivery at 35 weeks and 6 days’ gestation, with a birth weight of 2,800 g. Her Apgar scores were 10 at both 1 and 5 min. She was breastfed after birth with normal stool frequency and volume. One day prior to admission, she developed abdominal distension that progressively worsened and was accompanied by vomiting and a reduction in spontaneous defecation. On admission, her temperature was 36.3℃, heart rate 148 bpm, respiratory rate 70 bpm, and blood pressure 90/47 mmHg. The patient exhibited markedly poor peripheral perfusion, with a capillary refill time of approximately 4 s, suggestive of compromised circulatory status. The patient appeared lethargic and pale, and the abdomen was markedly distended and tense. Respiratory effort was increased, with visible inspiratory retractions, indicating significant respiratory distress. Laboratory test findings revealed: white blood cell count (WBC) 37.3 × 10^9^/L, neutrophil percentage (*N*%) 42.4%, hemoglobin (Hb) 6.6 g/dL, and C-reactive protein (CRP) level 4.3 mg/L. The arterial blood gas analysis showed pH 7.115, PCO₂ 18.4 mmHg, PO₂ 38.9 mmHg, Na + 132 mmol/L, K + 6.3 mmol/L, Lactate 21 mmol/L, and Base Excess −21.6 mmol/L. Abdominal x-ray, as shown in Figure 1, and computed tomography (CT) examination revealed significant gaseous distension of abdominal bowel loops with multiple step-ladder air-fluid levels in the right upper quadrant, which were suggestive of a low intestinal obstruction. The abdominal ultrasound also showed dilated and aperistaltic intestinal loops with a thinned bowel wall and poor perfusion, and multiple anechoic fluid collections in the abdominal cavity. Initial management included fasting, gastrointestinal decompression, invasive mechanical ventilation, correction of acidosis, fluid resuscitation, inotrope/vasopressor infusion, antibiotic therapy with cefoperazone- sulbactam and blood transfusion. Subsequently, diagnostic ascitic paracentesis was performed to characterize the fluid, yielding 57 mL of hemorrhagic ascites. Clinically, the worsening abdominal distension was accompanied by bloody stools, increased abdominal wall tension, and absent bowel sounds. Given these findings, emergency exploratory laparotomy was immediately performed. Marked dilatation of the small intestine was observed intraoperatively, starting approximately 70 cm distal to the ligament of Treitz, with a paper-thin bowel wall exhibiting a dusky purple discoloration. This indicated probable intestinal ischemia and necrosis, which extended to within 10 cm of the ileocecal junction, with the necrotic segment measuring approximately 26 cm, as shown in Figure 2. The mesenteric root showed a 360° counterclockwise volvulus. An ileostomy was created 70 cm distal to the ligament of Treitz. The hematoxylin and eosin examination showed preservation of gross architecture with mucosal necrosis, congestion, and thrombosis within the mucosal layer, as shown in Figure 3. Therefore, a definitive diagnosis of PSV was made postoperatively. Preoperative coagulation assessment: Prothrombin time (PT) was 15.8 s and activated partial thromboplastin time (APTT) was 54.1 s. Antithrombin III (AT III) activity was 51%, fibrin/fibrinogen degradation products (FDP) were 20.90 μg/mL, and D-dimer was 5.48 mg/L FEU. Platelet count was 445 × 10^9^/L. Thrombin-antithrombin complex (TAT) was 42.0 ng/mL. The preoperative coagulation findings reflect activation of the coagulation system, but do not meet the criteria for disseminated intravascular coagulation (DIC). Notably, the preoperative TAT level normalized rapidly postoperatively to 2.7 ng/mL (normal range <4.0 ng/mL). Blood and ascites cultures were negative. However, metagenomic next-generation sequencing (mNGS) performed on both blood and ascitic fluid detected identical pathogens, including Escherichia coli, Clostridium difficile, and Enterococcus faecalis. Antibiotic therapy was adjusted according to microbial susceptibility profiles and continued for 14 days. The patient remained on invasive mechanical ventilation from admission until postoperative day 7, which ensured adequate oxygenation and respiratory stability during the acute and perioperative phases. The patient was discharged in good condition after a 15-day hospital stay. Stoma closure surgery was performed three months later. During the period with the ileostomy, the infant didn't experience significant high output from the ileostomy, because of approximately 70 cm distal to the ligament of Treitz. She was discharged on full oral feeds, and the volume of stama output was less than 30 mL/kg/day. At present, the child is 1 year and 11 months old with normal growth and neurodevelopment without any further complications.

## Discussion

Volvulus is usually a severe and rapidly progressive condition with high destructive potential. Volvulus can be categorized into total midgut volvulus and segmental volvulus (SV) based on the length of the intestinal segment involved. For neonates, the most common clinical type is congenital intestinal malrotation with midgut volvulus; however, SV is relatively rare (2). A small subset of cases present as isolated SV without anatomical or pathological abnormalities, which is referred to as PSV (1, 3, 4). A previous study has shown that PSV usually occurs in preterm infants, particularly at two specific time points, with a median age of 3 days for early-onset volvulus and 45 days for late-onset volvulus. However, the corrected gestational ages of these patients are similar, suggesting that PSV can occur at specific gestational ages of neonates, which is consistent with the age of onset observed in the present case (5). Currently, the etiology of PSV remains unclear. On the one hand, the unique physiological structure of neonates leads to impaired peristalsis, which delays or even obstructs the emptying of intestinal contents (6). Moreover, the presence of immature ganglion cells in the intestinal tract also exacerbates this condition (7). On the other hand, the special anatomical structure of preterm infants, including the congenital mesenteric elongation or constriction of the small intestine, can increase the risk of developing PSV (8). In addition, non-invasive ventilation (NIV), especially nasal high-frequency oscillation (nHFO), has been suggested to promote intestinal dilation, potentially increasing the risk of PSV (9). Neonates typically present with the clinical manifestations of low small intestinal obstruction, including abdominal distension, bilious vomiting, and feeding intolerance. Notably, in infants presenting with acute abdomen, severe anemia, particularly in a 3-month-old, should be recognized as a critical warning sign, as it may indicate ongoing intestinal ischemia or necrosis. PSV is essentially a form of strangulated bowel obstruction, and, therefore, patients may also exhibit bloody stools, abdominal tenderness, ascites, and hypotension. As the condition progresses, metabolic acidosis, sepsis, shock, DIC, and even death can occur (10). An upper gastrointestinal (UGI) contrast study is generally the first-line imaging test for ruling out intestinal malrotation (11). However, in this infant, given the presence of severe abdominal distention and peritonitis with a risk of intestinal necrosis, UGI was avoided, as the hypertonic contrast agent might exacerbate bowel necrosis. Abdominal ultrasound findings may reveal nonspecific findings such as bowel dilation, bowel wall thickening, and ascites, whereas the “whirlpool sign” is rarely observed (12). It should be noted that abdominal ultrasonography, which is inherently operator-dependent, was performed by the attending radiologists in the Department of Ultrasonography or by our neonatologist in our neonatal intensive care unit (NICU). All of these clinicians have extensive experience in clinical and critical care ultrasound. An abdominal x-ray may exhibit features consistent with low intestinal obstruction. The presence of a fixed bowel loop, without signs of intestinal atresia—such as significant bowel dilatation, gasless abdomen, and “double-bubble sign” on multiple abdominal x-rays—may suggest PSV. Nevertheless, this imaging finding is non-specific and must be interpreted in clinical context. Furthermore, diagnostic paracentesis may yield bloody ascites, indicating bowel necrosis and further supporting the suspicion of PSV. In this case, rapid diagnostic paracentesis was performed to evaluate the characteristics of the ascitic fluid, assess for pneumoperitoneum, obtain a valuable sample for pathogen detection, and transiently relieve intra-abdominal pressure. The infant had an uneventful feeding history prior to presentation, without signs of feeding intolerance, and presented with an abrupt onset of symptoms. Based on the clinical manifestations and imaging findings, PSV was suspected preoperatively and subsequently confirmed during surgery.

**Figure 4:**
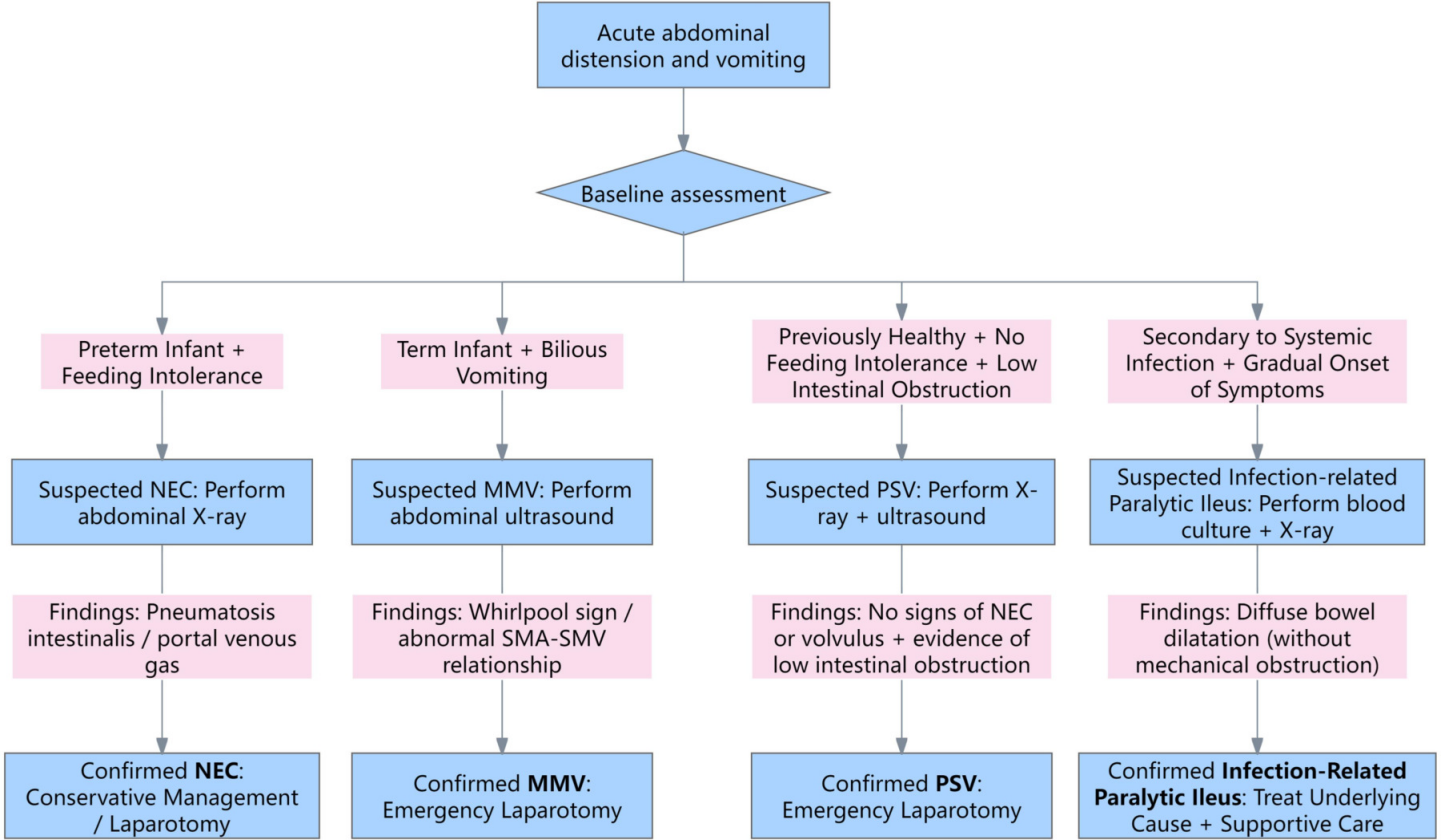
The differential diagnosis flowchart. NEC, necrotizing enterocolitis; MMV, malrotation with midgut volvulus; PSV, primary segmental volvulus; SMA, superior mesenteric artery; SMV, superior mesenteric vein.

Before proceeding to surgery, it was essential to consider other potential causes of acute neonatal abdominal distension. NEC is the most common surgical emergency in premature infants, typically presenting in the second to third week of life with feeding intolerance, bilious vomiting, abdominal distension, and blood in stool (13). Abdominal x-ray may show pneumatosis intestinalis, portal venous gas, or pneumoperitoneum (14). Intestinal malrotation with midgut volvulus also presents acutely with bilious vomiting and abdominal distension, but is characterized by specific ultrasonographic signs, such as the “whirlpool sign” and abnormal orientation of the superior mesenteric artery and vein (15). Infection-related paralytic ileus is a functional obstruction secondary to systemic infections such as sepsis, typically presenting with diffuse bowel dilatation without evidence of mechanical obstruction, and a more gradual onset of symptoms (16). In contrast, PSV typically occurs in previously healthy neonates, presenting with bilious vomiting and abdominal distension without feeding intolerance. It can be differentiated from NEC, midgut volvulus, and paralytic ileus by manifesting as a low intestinal obstruction and lacking the characteristic radiologic or ultrasonographic features associated with these conditions. The differential diagnosis flowchart is shown in Figure 4.

PSV is a rare and severe condition, and timely and effective treatment is crucial to the prognosis of neonates. Surgical intervention is currently the main treatment for neonatal PSV because nearly all patients require operative management. However, making a definitive preoperative diagnosis is often difficult due to the rarity and nonspecific clinical presentation of PSV (1).Furthermore, Kim et al. suggests that ultrasound and UGI have limited diagnostic yield for PSV, indicating that the need for a high index of clinical suspicion and careful clinical assessment rather than reliance on imaging (1). Once diagnosed, emergency exploratory laparotomy should be performed promptly to prevent progression to sepsis, DIC, shock, and bowel necrosis, which may result in short bowel syndrome. PSV may lead to acute peritonitis, which can trigger DIC in neonates. TAT, formed by the binding of thrombin to antithrombin, serves as an early warning marker of coagulation activation, often rising before conventional coagulation parameters (17, 18). In our NICU, TAT measurement is routinely used to monitor coagulation status in critically ill patients (19). In this case, the patient's preoperative TAT level was markedly elevated at 42 ng/mL (normal range <4 ng/mL), reflecting systemic coagulation activation. Although TAT is a sensitive marker of coagulation activation, it is not specific for intestinal necrosis and is not unique to PSV; elevated levels can also be observed in other neonatal conditions such as sepsis or NEC (20, 21). In this case, at the time when both blood and ascitic fluid were collected for mNGS testing, concurrent blood tests showed WBC 2.6 × 10⁹/L, N% 76%, Hb 111 g/L, and CRP 230 mg/L, consistent with secondary bacterial infection. mNGS detected Escherichia coli, Clostridium difficile, and Enterococcus faecalis, typical gut flora, suggesting bacterial translocation from ischemic and necrotic bowel rather than contamination. In our NICU, we performed mNGS using precious body fluids such as ascitic fluid or bronchoalveolar lavage fluid complement blood sample to improve the probability of a positive diagnosis and optimize therapy for critically ill newborns (19). Despite its advantages, mNGS cannot replace traditional culture due to the absence of antimicrobial susceptibility testing results. These findings guided targeted antimicrobial therapy, with results generally available within 24–48 h, enabling timely adjustment of treatment.

Therefore, perioperative management should not only involve the use of mNGS to identify pathogens for targeted anti-infective therapy but also include the test of TAT to monitor coagulation status, with anticoagulant therapy considered when necessary.

## Conclusions

PSV is a rare and life-threatening cause of intestinal obstruction. It often lacks specific clinical and radiological signs, which makes early diagnosis challenging. Timely recognition and prompt surgical intervention remain crucial for improving patient outcomes and preventing severe complications such as bowel necrosis, short bowel syndrome, and DIC. TAT levels may serve as a useful marker for monitoring coagulation status in the perioperative period. Furthermore, the use of mNGS can help in the precise identification of pathogens and facilitate targeted antibiotic therapy. Overall, comprehensive perioperative management, including early surgical treatment, coagulation monitoring, and targeted antibiotic therapy, may be beneficial for improving clinical outcomes in PSV.

## Data Availability

The original contributions presented in the study are included in the article/Supplementary Material, further inquiries can be directed to the corresponding author/s.
